# Strategies of rational and structure-driven vaccine design for Arenaviruses

**DOI:** 10.1016/j.meegid.2024.105626

**Published:** 2024-06-20

**Authors:** Antonia Sophia Peter, Dieter S. Hoffmann, Johannes Klier, Christina M. Lange, Johanna Moeller, Victoria Most, Christina K. Wüst, Max Beining, Sevilay Gülesen, Hannes Junker, Birke Brumme, Torben Schiffner, Jens Meiler, Clara T. Schoeder

**Affiliations:** a Institute for Drug Discovery, Leipzig University, Faculty of Medicine, Leipzig, Germany; b Center for Scalable Data Analytics and Artificial Intelligence ScaDS.AI, Dresden/Leipzig, Germany; c Molecular Medicine Studies, Faculty for Biology and Preclinical Medicine, University of Regensburg, Regensburg, Germany; d SECAI, School of Embedded Composite Artificial Intelligence, Dresden/Leipzig, Germany; e The Scripps Research Institute, Department for Immunology and Microbiology, La Jolla, CA, United States; f Department of Chemistry, Vanderbilt University, Nashville, TN, United States; g Center for Structural Biology, Vanderbilt University, Nashville, TN, United States

**Keywords:** *Mammarenavirus*, Arenavirus, Vaccine, Computational vaccine design, Lassa virus

## Abstract

The COVID-19 outbreak has highlighted the importance of pandemic preparedness for the prevention of future health crises. One virus family with high pandemic potential are Arenaviruses, which have been detected almost worldwide, particularly in Africa and the Americas. These viruses are highly understudied and many questions regarding their structure, replication and tropism remain unanswered, making the design of an efficacious and molecularly-defined vaccine challenging. We propose that structure-driven computational vaccine design will contribute to overcome these challenges. Computational methods for stabilization of viral glycoproteins or epitope focusing have made progress during the last decades and particularly during the COVID-19 pandemic, and have proven useful for rational vaccine design and the establishment of novel diagnostic tools. In this review, we summarize gaps in our understanding of Arenavirus molecular biology, highlight challenges in vaccine design and discuss how structure-driven and computationally informed strategies will aid in overcoming these obstacles.

## 1. Arenaviruses

Vaccines are one of the safest, simplest, and most powerful tools for the prevention of viral diseases. One of the major obstacles for the development of effective vaccines is the oftentimes scarce knowledge on potential viral target proteins, especially in case of understudied viruses. One emerging option for the efficient development of novel vaccines are structure-driven protein design methods. These methods have made progress during recent years, supporting the field of vaccinology with a multitude of new strategies based in the fundamental research of structural biology of viral glycoproteins (Schoeder et al., 2022; Narykov et al., 2021; Wei et al., 2020a; Castro et al., 2022).

Arenaviruses form a virus family that is severely understudied but poses a significant threat to global health, especially the *Mammarenavirus* genus that infects mammals (Zhou et al., 2012). While asymptomatic infections with viruses from this family can occur in humans, some viruses can result in devastating or even deadly disease outcomes with case fatality rates of up to 80% (Radoshitzky and de la Torre, 2019). *Mammarenaviruses* are generally subdivided into old world (OW) and new world (NW) Arenaviruses depending on their geographical origin, with the former originating on the African continent and the latter being found in South and North America (Brisse and Ly, 2019) (Table 1). Deaths caused by these viruses are usually attributed to hemorrhagic fevers or aseptic meningitis. Viruses causing hemorrhagic fevers include Lassa virus (LASV), Lujo virus (LUJV), Junin virus (JUNV), Machupo virus (MACV), Guanarito virus (GOTV), Sabia virus (SABV), Chapare virus (CHAV) or Whitewater Arroyo virus (WWAV). Viruses causing aseptic meningitis include the Lymphocytic Choriomeningitis virus (LCMV) (Jason et al., 2010; Brisse and Ly, 2019; Briese et al., 2009). There are however, several *Mammarenaviruses* known, reviewed and listed in Brisse et al. such as the OW Mopeiva Virus (MOPV) and Mobala Virus (MOBV), for which the potential to cause pathogenic infection in humans remains unclear (Brisse and Ly, 2019). Infections in humans mainly result from zoonotic transmission from rodents living in close proximity to humans (Table 1) (Happi et al., 2022) but can also occur from human-to-human transmission, making Mammarenaviruses a significant threat for global health (Hallam et al., 2018a). *Mammarenaviruses* have high mutation rates allowing them to evade host immune responses and adapt to new receptors (Grande-Pérez et al., 2016; Markov et al., 2023). The segmented ambisense RNA (Ferrer et al., 2019; Martinez et al., 2013) genome mutates with a frequency of 2.6–5.5 × 10^−4^ mutations per nucleotide, which is approximately 100-fold higher than the mutation frequency of SARS-CoV-2 (1–2 × 10^−6^ mutations per nucleotide).

**Fig. 1.**
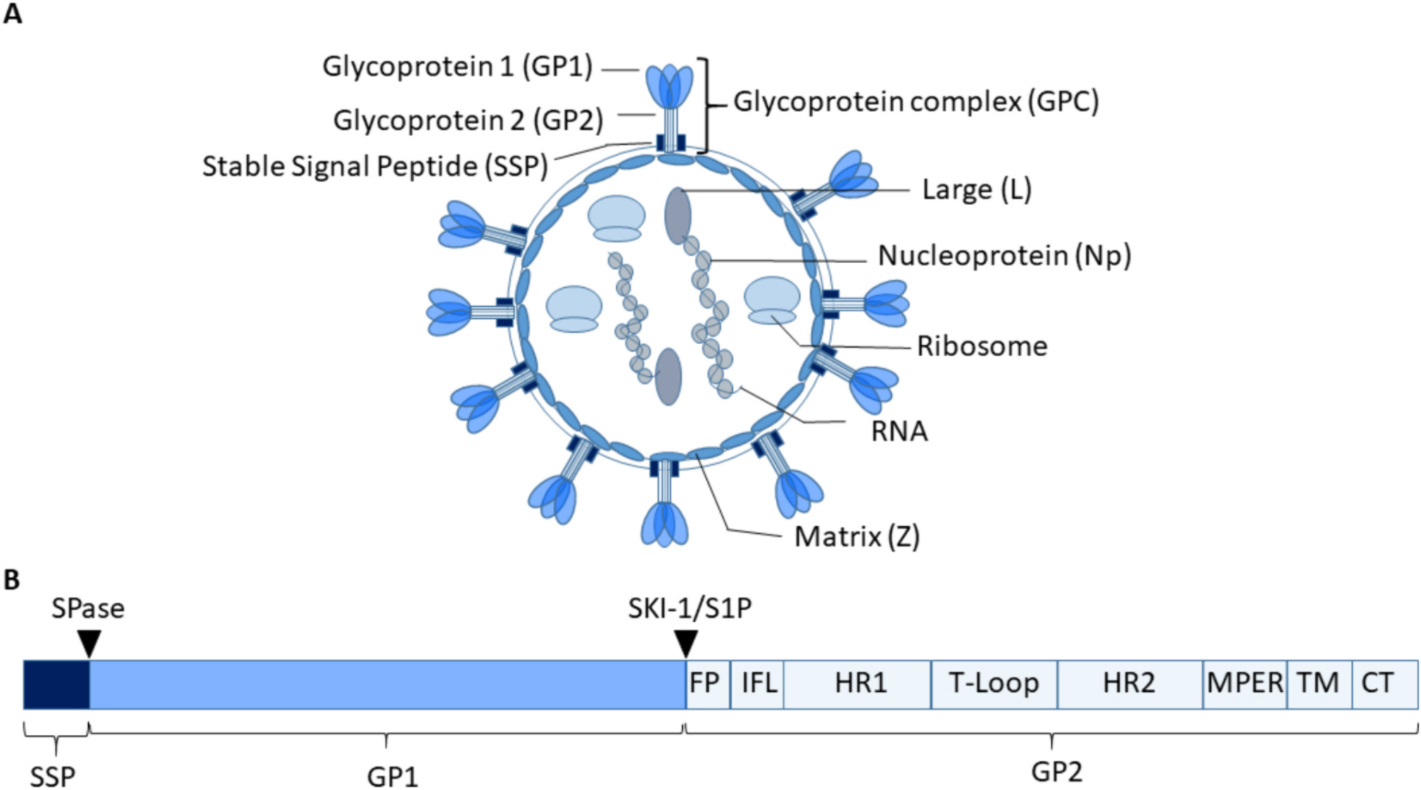
Arenavirus virion structure and Glycoprotein complex domains. Arenaviruses are enveloped viruses with a diameter of approximately 120 nm that contain two or three segments of single stranded RNA, a Large protein (L) that is an RNA-dependent RNA polymerase, a Matrix protein (Z) and the nucleoprotein (Np). Additionally, ribosomes that are of host cell origin are packaged into the virions (A). The only viral protein on the surface is the glycoprotein complex (GPC), comprised of the glycoprotein 1 (GP1) domain, the GP2 domain and the stable signal peptide. GPC is processed from a precursor protein through proteolytic cleavage, mediated by the signal peptidase (SPase) or subtilisin kexin isozyme-1 (SKI-1)/site-1 protease (S1P) (indicated by the triangles) (B). The GP1 domain contains the receptor binding motif whilst the GP2 domain contains a fusion peptide (FP), Internal fusion loop (IFL, two heptad repeat domains (HR1 and 2), a T-Loop domain that acts as a linker, a Membrane proximal external domain (MPER), a transmembrane domain (TM) and a C terminal domain (CT) that is located intracellularly. Not to scale (Garry, 2023; Pennington and Lee, 2022; Stott et al., 2020; Zhang et al., 2019).

Arenaviruses are enveloped viruses that bind receptors on macrophages or dendritic cells (Table 1). Viral entry is mediated by the glycoprotein complex (GPC), a class I transmembrane protein on the virion surface (Fig. 1A) (Stott et al., 2020). GPC is the sole viral surface protein, making it the only target for antibody-mediated neutralization (Brouwer et al., 2022). The GPC is comprised of glycoprotein 1 (GP1), GP2 and the stable signal peptide (SSP) (Katz et al., 2022; Nunberg and York, 2012) (Fig. 1). The latter is crucial for the transport and proteolytic maturation of the GP (Pennington and Lee, 2022; Stott et al., 2020; Joanne and Nunberg, 2006) (Cheng et al., 2015). The virion also contains a Matrix protein (Z) that is essential for viral budding, the RNA polymerase or Large protein (L) required for replication and Nucleoproteins (Np) that encapsulate the two or three segments of viral RNA and facilitate viral RNA generation (Garry, 2023) (Fig. 1A). The virion also contains host cell ribosomes, whose function remains to be determined (Gale et al., 2019).

## 2. The Arenavirus glycoprotein complex

The understudied nature of Arenaviruses becomes evident when regarding the GP, as its structural dynamics and flexibility are only rudimentarily understood. The GPC is expressed as a trimer of tripartite protomers on the viral surface (Brouwer et al., 2022). The GPC itself is composed of the GP1, GP2 and SSP domain (Fig. 1B). These domains are cleaved from a precursor polypeptide during viral maturation in the infected host cell. The SSP domain is cleaved off during post-translational modification by the signal peptidase (SPase) whilst the GP1-GP2 site is cleaved by the subtilisin kexin isozyme 1/site 1 protease (SKI-1/S1P) (Katz et al., 2022; Cohen-Dvashi et al., 2018; Kunz and de la Torre, 2017).

The SSP is comprised of 58 amino acids and it is assumed to have two membrane-spanning regions and a short ectodomain loop (Pennington and Lee, 2022; Joanne and Nunberg, 2016). Uniquely, the SSP remains associated with the GPC in Arenaviruses. For JUNV, the penultimate C-terminal cysteine of the SSP mediates the interaction with a zinc-binding domain within GP2 (Katz et al., 2022; Joanne and Nunberg, 2007). A similar observation was made for LCMV, where an “FLLL” sorting signal near the C-terminus seems to be responsible for GPC maturation and trafficking (Bederka et al., 2014). The SSP domain is thought to contribute to GPC maturation by masking endoplasmic retention signals on the GP2 domain (Joanne and Nunberg, 2006; Messina et al., 2012; Bederka et al., 2014). The exact interaction mechanism remains to be elucidated for other Arenaviruses but the C-terminal cysteine residue is conserved amongst OW and NW arenaviruses (Pennington and Lee, 2022). SSP was also shown to modulate pH-induced activation of membrane fusion, and mutations at residue 33 of the JUNV SSP lead to abrogated membrane fusion (Joanne and Nunberg, 2009). While this residue is conserved amongst Arenaviruses (Pennington and Lee, 2022), substitution of the JUNV SSP domain with another Arenavirus SSP inhibits GPC membrane fusion activity despite correct assembled and localization (Messina et al., 2012). Unusually, the N-terminus of SSP contains a myristoylation motif conserved amongst OW and NW Arenaviruses (Pennington and Lee, 2022), which has been shown to be merisoylated for JONV (Joanne et al., 2004). *N*-terminal myristoylation is a lipid modification that results in the addition of a myristoyl group to an *N*-terminal glycine residue and has been proposed to play a role JUNV membrane fusion (Joanne and Nunberg, 2016). Despite its importance for Arenavirus infectivity, little is known about the SSP domain (Joanne et al., 2008).

The GP1 domain contains a receptor binding domain (RBD), which acts as the primary interaction site for host cell. Although NW arenaviruses interact with Transferrin receptor-1 (TfR1), substantial differences in recognition of TfR1 and interacting residues could be observed, most likely due to varying TfR1 receptors across rodent reservoirs (Clark et al., 2018; Abraham et al., 2010). Several binding motifs have been postulated to contact TfR1 in different NW Arenaviruses (Abraham et al., 2010). For instance, Abraham et al. postulated a hydrogen bond between MACV S113 and TfR1 Y211 (motif 1) observed in crystal-structures (Abraham et al., 2010). However, this interaction is mediated by a Threonine in SABV or an Aspartate in JUNV whilst GOTV has a hydrophobic residue at the corresponding position and the contact is likely to be mediated by a proximal Glutamate (Abraham et al., 2010).

RBDs of OW Arenavirus are located at different sites within the GP1 domain and show a high degree of variability (Diskin, 2023), due to their interaction with diverse receptors (Table 1). The RBDs of LASV and LCMV are assumed to involve all three GP1 subunits, mainly interacting with the Matriglycan, a carbohydrate modification, of α-Dystroglycan (α-DG) (Katz et al., 2022). The RRLL residues of the neighboring promoters are packed against each other thereby forming a highly basic groove that mediates the interaction with the acidic matriglycan of α-DG (Katz et al., 2022; Moon-Walker et al., 2023). This interaction is enhanced by hydrophobic interactions formed between Tyr150 of one GP1 domain and Glu151 and Lys125 of the neighboring GP1 domain (Katz et al., 2022; Moon-Walker et al., 2023). The GP1 domain also contains the second RBD, such as the histidine triad of LASV (Pennington and Lee, 2022), which mediates the interaction of OW arenaviruses with their secondary receptor. The substantial differences in receptor recognition amongst OW and NW viruses make the development of a pan-Arenavirus vaccine a very challenging.

**Fig. 2.**
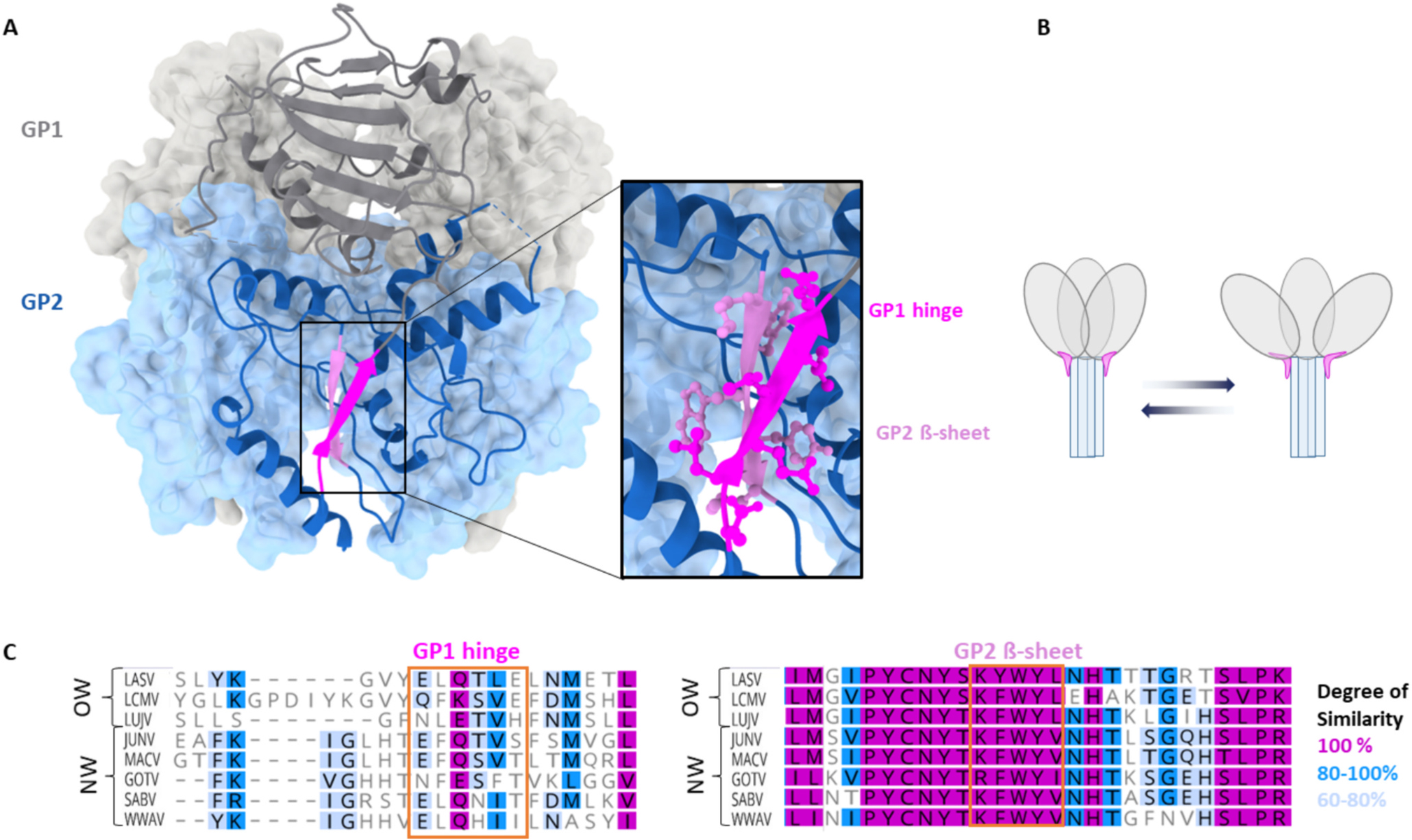
Structure and sequence conservation of the hinge region of Arenaviruses. The structure of GPC (PDB 7UL7) of the OW *Mammarenavirus* LASV is shown from the side (A), with an image detail displaying the interactions between the GP1 hinge and the backing GP2 β-sheet. (B) Schematic representation of the proposed breathing mechanism enabled by the hinge region shown in magenta. (C) Sequence alignment of the GP1 hinge region and the GP2 backing β-sheet located of the indicated viruses. The degree of similarity was determined using a Blosum62 matrix. The Alignment is based on the following PDBs or Uniprot sequences: LASV (7UL7) (Buck et al., 2022), LCMV (8DMI) (Moon-Walker et al., 2023), C5ILC1 (LUJV), D2CFR7 (JUNV), Q6IUF7 (MACV), Q8AYW1 (GOTV), Q90037 (SABV), and Q911P0 (WWAV).

The GP1 and GP2 domain are non-covalently linked with limited dimer stabilizing mutations described to date (Hastie et al., 2016; Moon-Walker et al., 2023; Gorman et al., 2024). The structure of LASV reveals a β-sheet motif of GP1, that is wedged into the GP2 domain (Fig. 2A). This motif is highly conserved amongst both OW and NW Arenaviruses (Fig. 2C). Interestingly, the GP2 β-strand interacting with this wedge is also conserved amongst *Mammarenaviruses* (Fig. 2B), suggesting an essential role of this motif. The positioning might allow for it to act as a hinge, giving the GP1 domain the needed flexibility for receptor binding. We hypothesize that this GP1 hinge might confer structural plasticity to the arenavirus GPC (Lewis et al., 1998; Medits et al., 2020; Stiasny et al., 2023; Casalino et al., 2022) (Fig. 2B), similar to the breathing behavior suggested for other class I fusion proteins such as the Influenza virus hemagglutinin (Amaro et al., 2018). The arenavirus hinge region forms known epitopes of LASV neutralizing antibodies, suggesting that these antibodies neutralize by locking the GPC in its prefusion conformation. It is likely, that mutations within the hinge, the backing β-sheet or the connecting loop, could alter protein stability and conformation, potentially facilitating vaccine design. In conclusion, many questions regarding the Arenavirus GPC function and structure remain unanswered.

## 3. Arenaviruses interact with diverse host receptors

For some Arenaviruses the reservoir and primary source of zoonotic infection remains unknown, posing a challenge for disease prevention (Table 1). In other cases, like LASV, new mammal reservoirs like rats have been described recently (Happi et al., 2022).

Moreover, for many Arenaviruses the attachment factors and ultimately also viral entry into the host cell are not fully understood. OW Arenaviruses utilize receptor mediated endocytosis while NW Arenaviruses employ clathrin-mediated endocytosis (Garry, 2023; Stott et al., 2020), both involving endosome acidification. Acidification of OW arenaviruses leads to protonation-mediated conformational changes in GP1 that ultimately results in a receptor switch due to a decreased affinity for the primary host cell receptor (Pennington and Lee, 2022; Zhang et al., 2019; Hadas et al., 2016; Li et al., 2016) (see Table 1). This process is investigated best for LASV where GP1 switches from binding to α-DG to binding to the lysosomal-associated membrane protein 1 (LAMP1). Binding to the primary receptor α-DG is only possible if matriglycan, an *O*-mannosylation, was attached to its mucin-type domain by LARGE (Katz et al., 2022; Stefan et al., 2005). Binding to the secondary LAMP-1 receptor is suggested to occur through protonation of GP1 which leads to the exposure of the histidine triad that interacts with LAMP1 (extensively reviewed in Pennington and Lee (Pennington and Lee, 2022)). This leads to shedding of GP1 and exposure of the GP2 subunit, which undergoes a conformational changes resulting in the exposure of the N-terminal fusion peptide (FP) and internal fusion loop (IFL) (Pennington and Lee, 2022). Interestingly, Arenaviruses have two separate fusion peptides, whereas most class I fusion proteins have a singular fusion peptide (Pennington and Lee, 2022; Rey and Lok, 2018). After the conformational change, the GP2 subunit can insert into the lysosomal membrane, resulting in a metastable pre-fusion hairpin. Subsequently, this structure rearranges into an energetically more favorable six-helix bundle (6HB) which enables the fusion of lysosomal and viral membranes and subsequent release of the viral genome into the cytosol (Pennington and Lee, 2022; Garry, 2023; Linero et al., 2018; Stott et al., 2020). Similar mechanisms have been proposed for NW Arenaviruses, but other reports suggest that a receptor switch is unlikely for NW Arenaviruses (Aliza et al., 2019). Pryce et al. showed, through a comparative study of NW and OW GPC crystal structures, that only OW GP1s form a distinct GP2-free state, characterized by an alternative orientation of the helical regions. Furthermore, NW viruses lack the required histidine triad (Rhys et al., 2018). To date, no secondary receptor of NW viruses has been identified and therefore further research is required to gain insight into NW entry mechanisms (Table 1) (Sarute and Ross, 2017).

Virus such as Coronaviruses, Flaviviruses or Retroviruses exploit cellular C-type lectins for cell attachment, entry and subsequent viral dissemination (Cambi et al., 2005; Geijtenbeek et al., 2000; Alvarez et al., 2002; Tassaneetrithep et al., 2003; Amraei et al., 2021). The surface glycoproteins of these viruses contain under-processed high-mannose glycans that interact with host lectins (Carbaugh and Lazear, 2020; George et al., 2003; Routhu et al., 2019). Interestingly, OW and NW Arenaviruses display mannosylated residues on their glycoproteins, making C-type lectins potential interaction partners (Geijtenbeek et al., 2000; Ana-Rita et al., 2013; Watanabe et al., 2018). Overexpression of C-type lectin receptor dendritic cell specific intercellular adhesion molecule-3-(ICAM-3) grabbing non integrin (DC-SIGN) or the liver/lymph node specific ICAM-3-grabbing non-integrin (L-SIGN) lead to an enhancement of NW Arenavirus infection, specifically for JUNV. A similar phenomenon was observed for the OW LASV: Lassa GP1 interacts with DC-SIGN via its *N*-terminal mannose residues which also facilitate entry (Ana-Rita et al., 2013), suggesting that C-type lectins can be involved in OW and NW Arenavirus cell entry (for overview see Fig. 2). Furthermore, when α-DG does not display optimal *O*-mannosylation, DC-SIGN and L-SIGN are utilized by LASV and LCMV (Watanabe et al., 2018).

Besides DC-SIGN and L-SIGN, Arenaviruses such as LASV, LCMV and some NW Arenaviruses interact with phospholipid phosphatidylserine (PtdSer) binding proteins like Tyro3/Axl/Mer (TAM) and T-cell immunoglobulin mucin I (TIM-1) (Masayuki et al., 2012; Brouillette et al., 2018; Sarute and Ross, 2021). These factors mediate entry by binding to host cell-derived PtdSer incorporated into the viral membrane during budding, making Arenaviruses behave like apoptotic bodies (Amara and Mercer, 2015). Alternative attachment factors of JUNV are not known, but it is likely that the virus also utilizes TIM and TAM due to membrane-incorporation of PtdSer. It remains to be determined whether DC-SIGN and L-SIGN might also act as receptors. Tan et al. observed that LUJV replication was significantly decreased in Niemann-Pick C1 (NPC1) deficient cells, suggesting its relevance for LUJV infection although the exact mechanism remains unknown (Hideki et al., 2014).

For NW, additional putative attachment factors have been identified. In case of JUNV and MACV, infection of mice with VGCC (voltage-gated calcium channels) knock-out lead to a decreased infection, potentially indicating VGCC as co-receptors or attachment factors. The exact mechanism, however, remains elusive (Lavanya et al., 2013). The decrease of infection could also be attributed to factors like a general decrease of Ca^2+^ in cells that is required for efficient pH-dependent viral membrane host cell fusion.

Although OW and NW Arenaviruses are capable of using alternative attachment factors for infection, viral entry via DC-SIGN was shown to be slower than via α-DG for LASV (Ana-Rita et al., 2013). Additionally, TAM overexpression was only able to rescue infection in the absence of α-DG (Chiara et al., 2018) but did not enhance infection with OW Arenaviruses in the presence of functional α-DG, indicating preference for primary receptor binding. Nevertheless, both OW and NW Arenaviruses using alternative attachment factors enhances zoonotic outbreak risks due to a broadening of their receptor spectrum, potentially contacting receptors with a high similarity between human and rodent.

The broad attachment factor spectrum also poses a significant challenge for vaccine design as not only the entry via the primary receptors needs to be blocked by the induced immune response but also the interaction with the attachment factors needs to be considered. Another challenge is that so far not all virus-host interaction mechanisms have been elucidated. The attachment to receptors like DC-SIGN or TAM and TIM could also pose a challenge for vaccine administration. If, for example, protein subunits are administered they might attach to DC-SIGN leading to protein degradation before an immune response to the antigen can mount. It could therefore be advantageous to employ new vaccination techniques like mRNA delivery that leads to localized protein expression in the individual and less protein dissemination (Fang et al., 2022). It has further been shown, that mRNA vaccination leads to a long lasting protein expression which could potentially contribute to a better immune response (Irrgang et al., 2022).

The fact that Arenaviruses seem to have different host-specific attachment factors prevents rational vaccine design from conveying vaccine breadth via the attachment factor binding site. Further studies will be needed to confirm or reject postulated attachment factor interactions and with this identify mechanisms of vulnerability for Arenaviruses.

## 4. *N*-linked glycans are crucial for Arenavirus pathogenicity

Viral surface glycoproteins are usually, as their name indicates, heavily glycosylated. The most common glycans in mammals are *N*-linked. These post-translational modifications are added during protein synthesis at conserved motifs characterized by N-X-S/T, with X being any amino acid but a proline (Li et al., 2021). Usually a high mannose core is attached which is then trimmed and modified as the protein is processed further in the endoplasmic reticulum and the Golgi (Li et al., 2021). This results in proteins with diverse oligosaccharides on their surface that can fulfill different functions, such as virus attachment, protection from host immune responses and modulation of infectivity or virulence (Li et al., 2021).

**Fig. 3.**
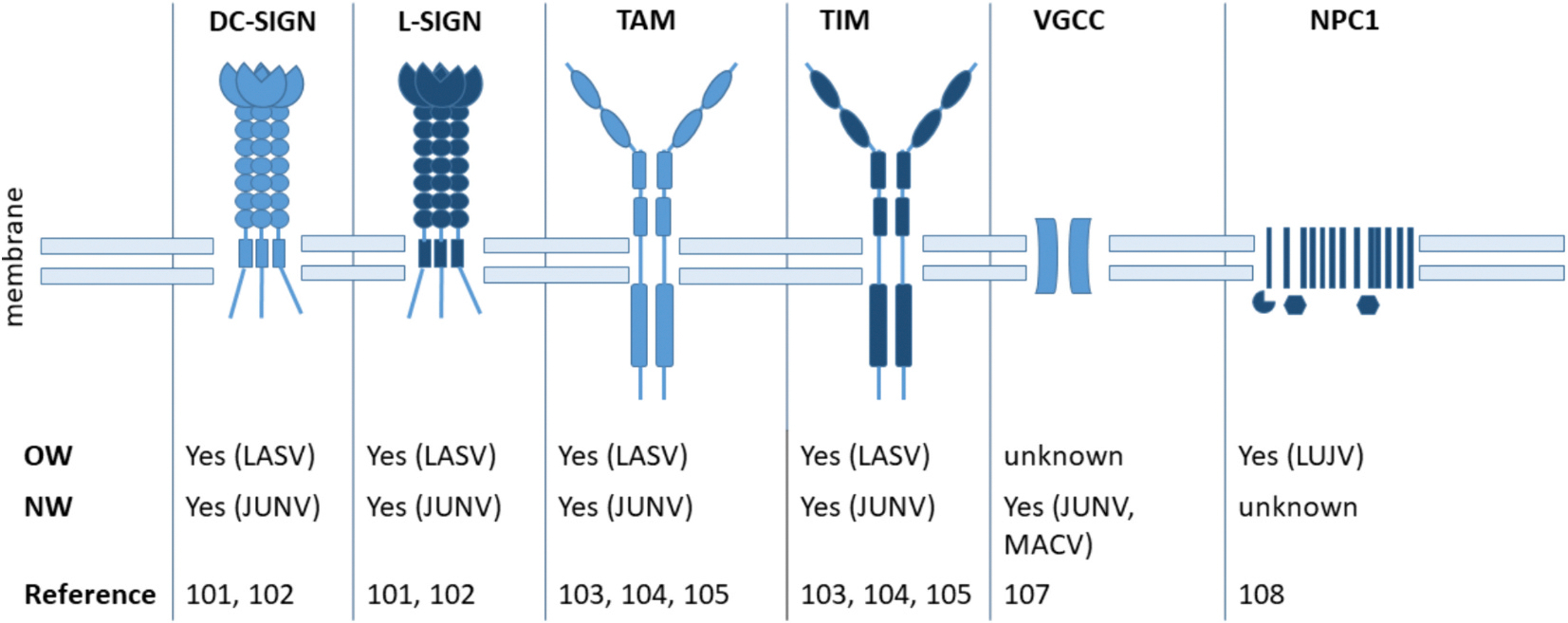
Putative attachment factors for OW and NW Arenaviruses. OW and NW Arenaviruses can use putative attachment factors for entering host cells. Shown are the respective factors as well as their usage by OW or NW viruses. Receptor dendritic cell specific intercellular adhesion molecule-3-(ICAM-3) grabbing non integrin (DC-SIGN), liver/lymph node specific ICAM-3-grabbing non-integrin (L-SIGN), Tyro3/Axl/Mer (TAM), T-cell immunoglobulin mucin I (TIM-1), voltage-gated calcium channels (VGCC) and Niemann-Pick C1 (NPC1).

**Fig. 4.**
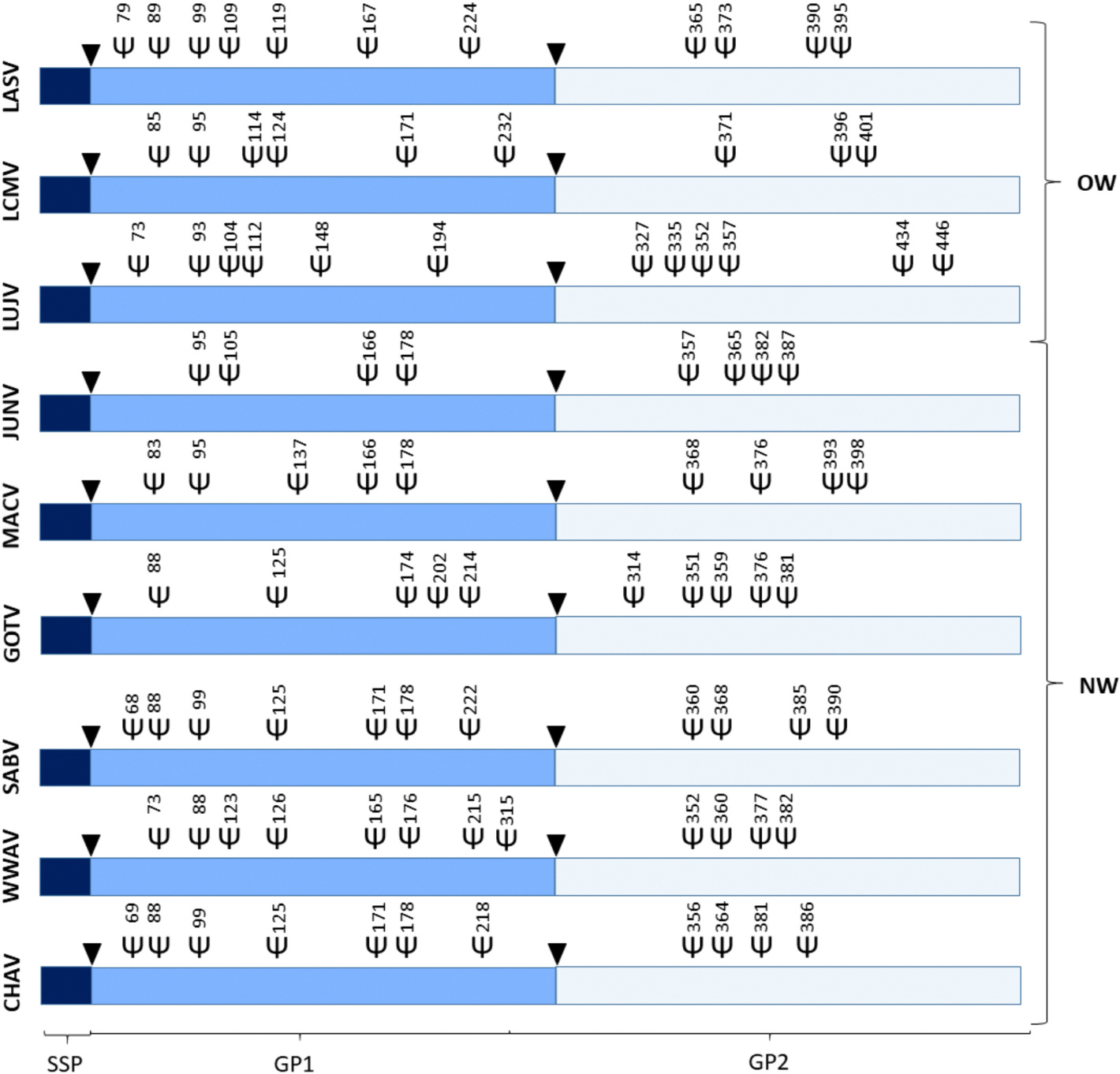
Arenavirus GPC glycosylation overview. Shown are the GPC precursor proteins of the Old World (OW) Lassa Virus (LASV), Lymphocytic choriomenengitis virus (LCMV) and the New World (NW) Lujo Virus (LUJV), Junin Virus (JUNV), Machupo virus (MACV), Guanarito Virus (GOTV), Sabia Virus (SABV), White Water Arroyo Virus (WWAV) and Chapre Virus (CHAV) with their respective predicted glycosylation sites within the SSP (Stable Signal Peptide), GP1 (Glycoprotein 1) and GP2 (Glycoprotein 2). The black arrows indicate proteolytic cleavage sites. Glycosylation predictions were made based on the following sequences Uniprot LASV (P08669), LCMV (P09991), C5ILC1 (LUJV), D2CFR7 (JUNV), Q6IUF7 (MACV), Q8AYW1 (GOTV), Q90037 (SABV), Q911P0 (WWAV) and GeneBank EU260463.1 (CHAV) with the NetNGlyc - 1.0 tool that predicts *N*-Glycosylation sites by examining the sequence context of N-X-S/T sequons using artificial neural networks (Gupta and Brunak, 2002). Not to scale.

Arenavirus GPCs are heavily glycosylated especially within the GP1 domain. Here, glycan positioning and frequency varies dramatically between different species (Fig. 3). The GP2 subunit is less glycosylated across all *Mammarenaviruses*, most having four predicted *N*-linked glycosylation sites (Fig. 3) (Bonhomme et al., 2011). The GP1 subunit, is as previously discussed, the primary interaction site between the virus and the host cell. It is therefore also a major point of vulnerability and oftentimes the primary target for host immune responses. One of these responses are neutralizing antibodies that usually either inhibit binding of the virus to the host cell or hinder conformational changes essential for viral entry. Glycosylation can protect the virus from these antibodies by forming the so-called glycan shield, that obscures potential antibody binding sites (Watanabe et al., 2020). This is in accordance with the observations made by Sommerstein et al., who saw the impairment of the neutralization capacity of LASV-specific antibodies by glycosylation (Sommerstein et al., 2015). They described, that the glycosylation motif at N119 of LASV conceals the α-DG binding site, making it inaccessible for neutralizing antibodies. Thus antibodies neutralize LASV variants lacking glycans more efficiently (Sommerstein et al., 2015). This could also be the reason why such low neutralizing antibody levels are observed after LASV infection in patients or GP1 immunization in mice (Brisse and Ly, 2019; Aliza et al., 2019; Sommerstein et al., 2015). This contrasts with the antibody responses observed in JUNV infected patients. Here, routinely robust neutralizing antibody levels can be measured that can also be replicated by GP1 immunization in mice (Aliza et al., 2019; Mantlo et al., 2019; Enria et al., 2008). This observation could potentially be attributed to the fact that JUNV GP1 Is less glycosylated than LASV GP1 thereby fewer neutralizing epitopes might be concealed, offering attack points for antibody mediated neutralization. Nevertheless evasion from neutralization mediated by glycosylation of the JUNV GP1 has also been described (Koma et al., 2021). Interestingly, low or delayed neutralizing antibody responses were also observed in LCMV infected patients. LCMV has, like LASV, a relatively heavily glycosylated GP1 subunit (Fig. 3). This indicates, that neutralizing antibody responses can predominantly be found in individuals infected with Arenaviruses that display relatively low GP1 glycosylation levels (Fig. 4). This, however, is hard to confirm due to the scarcity of data especially for the rarer Arenaviruses like WWAV, GOTV or CHAV.

The glycosylation of the GPC, however, not only influences the elicitation of monoclonal neutralizing antibodies but also the infectivity of the virus. As mentioned earlier it has been observed for LASV that N89 shields the LAMP1-binding site (Pennington and Lee, 2022). It is therefore likely that a loss of this *N*-glycosylation site might lead to a decrease in virulence. Moreover, LASV displaying aberrant glycosylation patterns was less infectious, likely because α-DG binding was also impaired. The exact mechanism how α-DG-GPC binding is influenced by glycans is not resolved yet (Punya et al., 2016). It could further be shown, that the removal of the glycans N79, N89, N365 and N373 in LASV lead to a decreased GP1/GP2 cleavage that in turn led to low pseudovirus titers (Li et al., n.d.). Nevertheless, a similar phenomenon was described for LCMV, almost all GPC functions were impaired if its glycosylation sites T87, S97 and G104 were mutated (Bonhomme et al., 2011). In case of MACV, the absence of *N*-linked glycans at N83 and N166 led to a partial attenuation of the virus in mice but no reduction in growth in cell culture could be observed. The in vivo attenuation was rescued by *N*-linked glycans at these positions (Koma et al., 2021). It was observed in this study, that the enhanced virulence in vivo was likely due to N83 and N166 either inhibiting antibodies from blocking sterical changes essential for virus entry, or by sterically hindering antibody access to the RBD, respectively (Koma et al., 2021). Position N166 is also glycosylated in JUNV but its absence does not seem to influence virulence in new born mice (Fig. 3) (Albariño et al., 2011). The loss of this glycosylation did, however, lead to the accumulation of GPCs as dimers and trimers that are more readily degraded in lysosomes (Manning et al., 2020; Manning et al., 2017). This makes antigen presentation more likely leading to higher immune responses. Further, it was observed that the elimination of N89 and N99 of LASV led to a reduction in GPC expression and abrogation of cleavage (Bonhomme et al., 2011). These findings highlight, that GPC glycosylation also has an influence on protein folding and epitope presentation having important implications for the design of vaccines against Arenaviruses.

## 5. Antibodies targeting Arenaviruses delineate points of vulnerability

As mentioned previously OW Arenaviruses, like LASV target dendritic cells and macrophages causing a generalized immune suppression leading to a tolerogenic T cell response (Murphy and Ly, 2022). It has been observed before, that this tolerance enhances disease severity, making robust T cell responses in Lassa-infected individuals an indicative marker for a favorable disease outcome (Ly, 2017; Hallam et al., 2018b). Surprisingly, B cell mediated responses have been shown to be dispensable for viral clearance (Murphy and Ly, 2022). Consequently, a lack of high neutralizing antibody titers has been observed in patients that recovered from acute LASV infections (Brisse and Ly, 2019; Sommerstein et al., 2015). Interestingly, Ronk et al. were able to observe, that guinea pigs were protected from a lethal LASV dose after mRNA LASV vaccination even though most animals only developed low neutralizing antibody responses and some even failed to mount a neutralizing response altogether (Ronk et al., 2023). This phenomenon could not be observed in the context of NW Arenaviruses which generally also target dendritic cells and macrophages (Sarute and Ross, 2017; Mantlo et al., 2019; Hallam et al., 2018b).

**Fig. 5.**
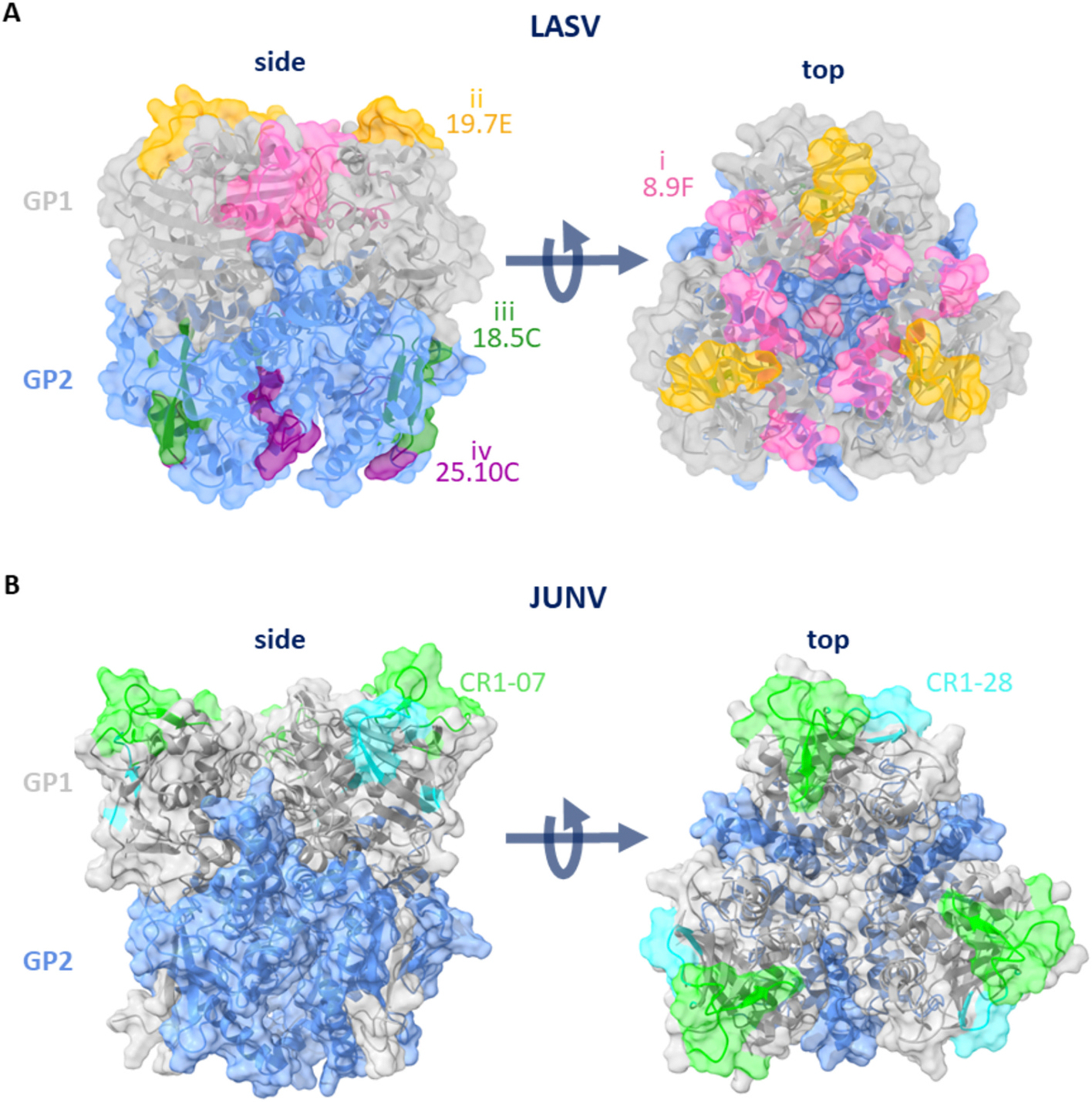
LASV residues contacted by 18.5C, 19.7E, 25.10C and 8.9F and JUNV residues contacted by CR1–28 and CR1–07. Shown are the structures of the LASV (PDB 7UL7) (A) and JUNV (B) from the side and the top. The residues contacted by the antibodies indicated are colored correspondingly. JUNV was modeled based on P26313 (Uniprot) with ColabFold (Mirdita et al., 2022). The GPCs were visualized using ChimeraX (Pettersen et al., 2004).

Nevertheless, only a limited number of studies investigating neutralizing epitopes of OW and NW Arenaviruses are available (Robinson et al., 2016). Robison et al. isolated mAbs from LASV fever survivors and found that the neutralizing antibodies can be categorized into four groups: antibodies targeting (i) the GPC apex formed by three GP1 subunits where the α-DG binding site is located, (ii) the GP1 region that forms the LAMP-1 binding site, (iii) the site across two GP2 protomers and GP1 located between glycans N390 and N395 and N79 and N99 and (iv) the region that crosses the GP1 and GP2 subunits and is delineated by glycans N79, N89, N99, N224, N365 and N373 (Li et al., n.d.). Interestingly, the majority of the LASV neutralizing human monoclonal antibodies (mAb) target epitopes that require both the GP1 and GP2 subunit thereby belonging either to group iii or iv (Robinson et al., 2016) (Supplement, Table 1, Fig. 5A, B). Antibody 18.5C, which belongs to group iii and is likely to bind holes within the glycan shield of GP1 and the fusion-loop or the T-loop of GP2 for example blocks infection by locking the GPC in its prefusion conformation (Buck et al., 2022; Robinson et al., 2016; Hastie et al., 2019). An example for an antibody that belongs to group iv is 25.10C. This group of antibodies is suggested to mediate neutralization by blocking LAMP1 binding and membrane fusion (Li et al., n.d.; Enriquez et al., 2022). Interestingly, both antibody groups, iii and iv, interact with residues that are located within the hinge region of GP1, possibly also locking the GP1 in a conformation that is inaccessible for the receptor. The antibody LAVA01, isolated from an LASV immunized rabbit, also targets epitopes on the GP1 and GP2 subunit but seems to belong neither to group iii or group iv. Since it interacts with residues that form the LAMP-1 binding site and it also contacts several glycosylation sites like N109 and N390, showing that glycans can not only mask antibody binding sites but also serve as targets (Supplement, Table 1) (Brouwer et al., 2022). Antibody 19.7E that belongs to group ii and is also unable to neutralize LASV when glycans at positions N109 or N167 are removed and blocks LAMP-1 binding (Supplement, Table 1, Fig. 5A, B) (Li et al., n.d.; Robinson et al., 2016) (Li et al., n.d.). Neutralization mediated by 12.1F (ii) is also reduced, when the glycosylation at position N109 is removed. As shown by Li et al., not only the presence of glycans at this position is important but also that they are of mammalian origin (Li et al., n.d.). This argues in favor of an mRNA vaccine, that bypasses the glycosylation of the antigen in a foreign host and ensures a glycosylation pattern native to the host. This argument is further supported by the fact, that group i antibodies are only able to interact with unfixed GPCs, making the elicitation of group i antibodies after the administration of an β-propiolactone inactivated virus unlikely (Li et al., n.d.). So far only one antibody that belongs to group i has been described, 8.9F (Table 1, Fig. 5A, B). This antibody targets a complex quaternary epitope encompassing both, GP1 and GP2 of all three protomers (Li et al., n.d.; Robinson et al., 2016; Enriquez et al., 2022). It is further characterized by an unusually long heavy chain complementarity-determining region 3 (HCDR3) region enabling these contacts (Robinson et al., 2016). This antibody fails to neutralize in the absence of N119 and also depends on the presence of mammalian glycosylation patterns (Li et al., n.d.).

Although the neutralization mechanism of the different anti-LASV antibodies is relatively similar their neutralization breadth, regarding the different LASV strains varies largely (Supplement, Table 1). Hence epitope variability needs to be taken into consideration during vaccine development. In comparison to other viruses there are only few antibodies known and the isolation of novel anti-LASV antibodies is necessary for a full description of the LASV humoral immune response. Nevertheless, the four antibody groups delineate points of vulnerability within the GPC of LASV that should be explored and preserved during vaccine development. It is of special interest to elicit antibodies that target more than one site of vulnerability like class iii and iv antibodies. These are more likely to withstand escape mutations and not only block receptor binding like class ii antibodies but interfere with structural changes of the GPC required for infection. It would also be interesting to investigate, whether the same points of vulnerability can be found in other OW Arenaviruses. Unfortunately, there are almost no antibodies described that target LUJV or LCMV (Supplement, Table 1). It could be shown that an engineered version of antibody 18.5C is able to neutralize LCMV, making it likely that similar sites of vulnerability can be found on this virus (Moon-Walker et al., 2023).

Although more NW than OW Arenaviuses that are pathogenic to humans could be identified to date, only few NW neutralizing antibodies have been described (Supplement, Table 1). Only two of these are of human origin, CR1–07 and CR1–28, and directed against JUNV and MACV. These two antibodies were isolated from an individual that received the live attenuated Candid #1 JUNV vaccine (Clark et al., 2018). Hence much information regarding the composition of the antibody response are lacking. Although antibodies isolated from mice can be useful, for diagnostic and therapeutic applications, antibody development in mice is characterized by a different somatic hyper mutation rate and most importantly shorter CDRs (Shi et al., 2014; Milholland et al., 2017). The latter in turn means, that antibodies like 8.9F are unlikely to be isolated from mice.

Most antibodies identified to neutralize JUNV or MACV act as TfR1 mimicry by inserting a tyrosine into the RBD that mimics the TfR1 Y221 thereby occupying the RBD (Clark et al., 2018). As could be shown for the JUNV neutralizing antibodies GD01 or OD01, they are highly strain specific which is likely linked to the different RBD interactions with the TfR1 receptor orthologues (Supplement, Table 1, Fig. 5C, D) (Sanchez et al., 1989). Interestingly, CR1–28 is able to weakly cross-neutralize MACV due to its different angle of approach (Clark et al., 2018). The other antibody isolated by Clark et al., CR1–07, does not interact with Y211 but contacts I115 and V117 on the rim of the RBD, thereby sterically blocking the RBD-TfR1 interaction (Supplement, Table 1). These residues have been identified to be the only two conserved residues between MACV and JUNV within the RBD (Clark et al., 2018). The MACV neutralizing antibody MAC1 has a different neutralization mechanism altogether, contacting peripheral residues located within loop 10 that can only be found in the GPC of MACV (Ng et al., 2022). Although the few NW neutralizing antibodies delineate some sites of vulnerability it is likely that many still remain to be identified. Consequently, there is an urgent and immediate need for the extensive characterization of the immune response against NW Arenaviruses.

## 6. Antibody dependent enhancement (ADE)

Since the primary target of Arenaviruses are antigen presenting cells like dendritic cells, such as monocytes and macrophages the question arises whether antibody dependent enhancement (ADE) plays a role during infection (Sarute and Ross, 2017; Hallam et al., 2018b). ADE has predominantly been described for Flaviviruses or the respiratory syncytial virus where the presence of virus specific antibodies exacerbates viral infection (Kosuke et al., 2022). This phenomenon is generally categorized into two different types, depending on the mechanism that is involved. ADE can occur either through enhanced immune activation, here predominantly non-neutralizing antibodies form immune complexes resulting in excessive Fc-mediated effector functions and immune cell activation, or through enhanced infection. Enhanced infection is mainly observed in macrophage-tropic viruses and caused by non- or sub-neutralizing antibodies that enhance viral endocytosis into macrophages via the Fc gamma receptor IIa ultimately resulting in enhanced viral replication in the target cells (Lee et al., 2020). To the knowledge of the authors no ADE has been described in the context of Arenaviruses, this could be due to the severely understudied nature of Arenaviruses or their geographical isolation from each other. The latter is likely to change due to the increasing globalization. The possibility of ADE arising during infection or after vaccination should therefore be taken into consideration and controlled carefully, especially as Arenaviruses have a significant amount of antigen overlap. This way weakly neutralizing antibodies to one Arenavirus strain could act as sub- or non-neutralizing antibodies to another Arenavirus strain enhancing viral infection. CR1–28, a strong JUNV and weak MACV neutralizer, or the KL-AV-2A1 antibody that binds to a conserved region on the GP2 of most Arenaviruses could potentially induce ADE (Clark et al., 2018; Fatima et al., 2018, Fatima et al., 2020). In summary, the potential development of ADE in vaccinated or infected individuals upon secondary contact with Arenaviruses needs to be considered.

## 7. Strategies for rational and computational vaccine design

### 7.1. Prefusion stabilization

Many details regarding Arenaviruses remain elusive and make the development of effective vaccines challenging. The main areas requiring further insight are determination of the Arenavirus reservoir range and its cellular receptors, mechanistic understanding of neutralization activity of antibodies, and with that the understanding of desired protective effects and vaccine side effects and safety. The latter is crucial as safety concerns arose regarding the stability of the attenuation phenotype of the JUNV Candid#1 vaccine licensed in Argentina (Gowen et al., 2021). Although it could be shown that a second-generation Candid#1 vaccine containing a K33S mutation leads to a more stable attenuation and also elicits robust antibody responses in guinea pigs (Gowen et al., 2021).Whilst a recombinant vesicular stomatitis virus vector based vaccine expressing the GPC of LASV lineage IV proved to be highly effective in non-human primates and is currently being tested in a phase II clinical trial (Cross et al., 2022; A Lassa Fever Vaccine Trial in Adults and Children Residing in West Africa, 2024). Concerning pandemic preparedness, rapid vaccine design for Arenaviruses will be the determinant of general rules for rational vaccine design across multiple lineages, and ideally multiple viruses. A discussion, that we are not including here, are suitable animal models, already established vaccine candidates or downstream vaccine validation experiments – these were reviewed extensively by Hastie et al. and Saito et al. (Saito et al., 2023; Hastie et al., 2023).

Fundamental understanding of the GPC structure is required for vaccine design. It could be shown for other class I fusion protein viruses, that arrest in the prefusion conformation, mediated for example by antibodies, leads to an abrogation of infection (Zhou et al., 2023). Hence, a vaccine that elicits this group of antibodies is desirable. This strategy has also successfully been employed in the context of different viruses including SARS-CoV-2 and RSV (Hastie et al., 2016; Hsieh et al., 2020; McLellan et al., 2013; Sanders et al., 2013; Rutten et al., 2020; Kulp et al., 2017; Juraszek et al., 2021; Dauparas et al., 2022). Classical approaches for prefusion stabilization are the strategic introduction of prolines, disulfides, other stabilizing mutations interface stabilization and introduction of multimerization domains. In all cases, computational protein design can assist traditional approaches through systematic search of suitable positions using multiple strategies.

The introduction of prolines can lead to a retention of GPCs in their prefusion conformation. This was achieved for SARS-CoV-2 by the introduction of six prolines into the Spike 2 domain that is comprised of several alpha helices that act similarly to the LASV GP2 domain (Hsieh et al., 2020). The strategy has also been proven successful for LASV, where the introduction of E329P into the HR1 of GP2 had a stabilizing effect, these mutations were introduced by rational vaccine design (Hastie et al., 2017). We therefore predict, that the strategic introduction of prolines, as predicted by computational proline screens, into the GP2 domain of other Arenaviruses will also lead to enhanced prefusion stability or even increased stability compared to the LASV E329P construct (Dauparas et al., 2022).

Another approach for the stabilization of viral glycoproteins is the introduction of disulfide bridges. The fusion glycoprotein from RSV subtype B DS-Cav1, for example, was stabilized in addition to cavity filling mutations by the introduction of a disulfide bridge that locks the protein in its prefusion conformation (McLellan et al., 2013; Joyce et al., 2019). A similar strategy was employed for LASV, where the introduction of disulfide bridges had a stabilizing effect (Gorman et al., 2024; Hastie et al., 2017). These inhibit the disassembly of the protein domains. Additionally, inter-protomer disulfides can be introduced to engineer trimeric proteins. Gorman et al. showed that immunization of guinea pigs first with trimer stabilized LASV GPC and then nanoparticles leads to robust immune responses (Gorman et al., 2024). Whether these mutations also have a stabilizing effect on other arenavirus GPCs remains to be determined (Hastie et al., 2016; Fleishman et al., 2011; Yao et al., 2022).

Viral glycoproteins can further be stabilized by the introduction of interface stabilizing and cavity filling mutations. As mentioned previously the DS-Cav1 construct was stabilized in addition to disulfide bridges by the introduction of cavity filling mutations. These substitutions increased the van der Waals contacts within the protein, increasing stability of the prefusion conformation (McLellan et al., 2013). This approach remains to be explored in the context of Arenaviruses. To this end the more recently described deep learning neural networks like ProteinMPNN (Dauparas et al., 2022), ESM (Lin et al., 2023) or Masked inverse folding with sequence transfer (MIFST) (Yang et al., 2023) could contribute to the design of a prefusion stabilized GPC as they consider amino acid substitutions based on naturally occurring structures or sequences.

Another option is the addition of artificial trimerization sites or the elimination or alteration of protein cleavage sites. The latter strategies have been used for the stabilization of the LASV GPC, where the introduction of a trimerization domain and the introduction of a furin site instead of the S1P cleavage site lead to improved stability and expression (Hastie et al., 2016; Brouwer et al., 2022). Trimerization domains like the foldon T4-fibritin, are known to elicit a strong and undesired immune response in vivo this can be avoided through the strategic introduction of glycans into the foldon that mask immunogenic domains (Sliepen et al., 2015).

### 7.2. Epitope focusing and glycan masking

**Fig. 6.**
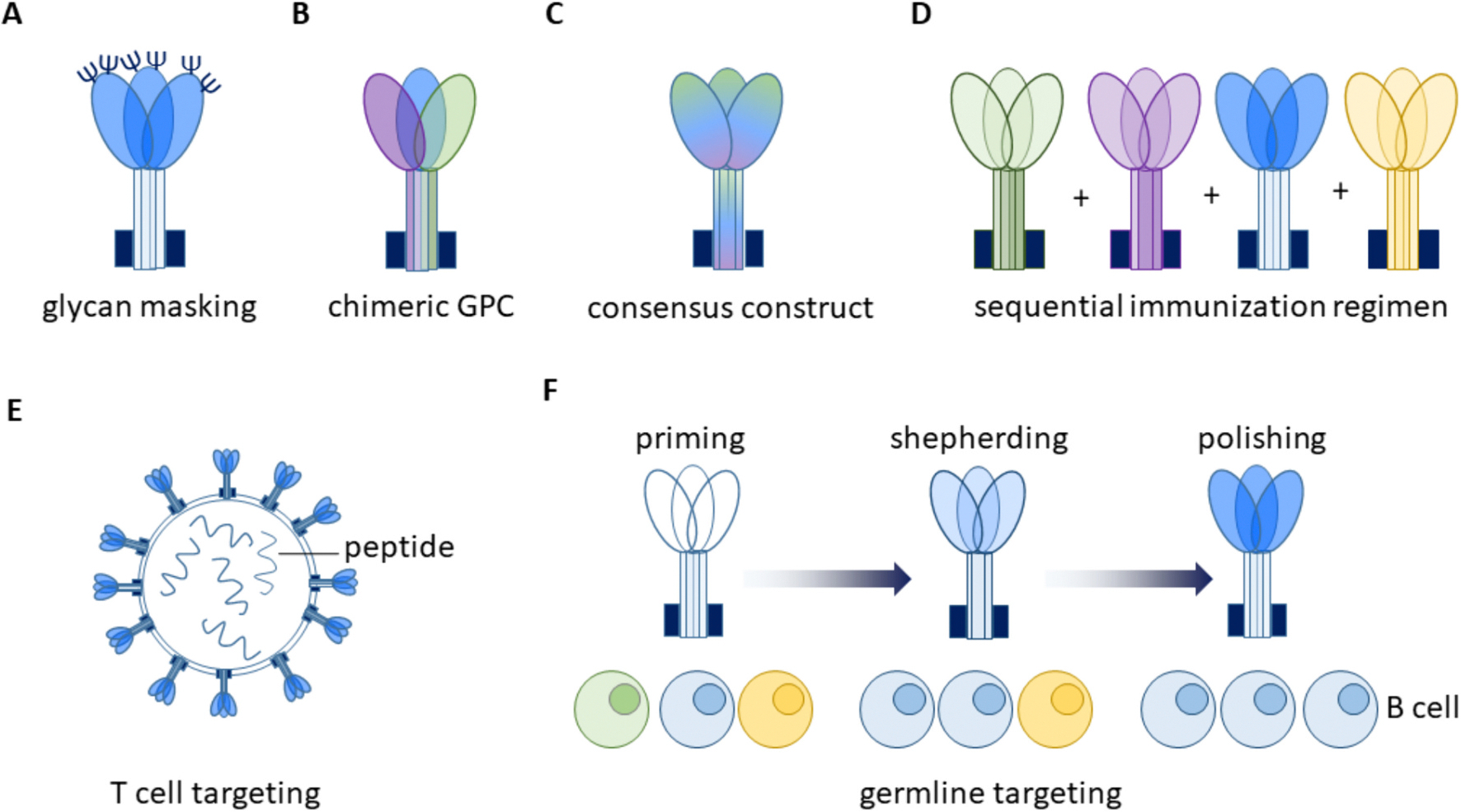
Strategies for a pan-Arenavirus vaccine generation. Several strategies can be employed for a pan-Arenavirus vaccine. Such as glycan masking of unwanted epitopes (A), the generation of a chimeric GPC, that is comprised of several different Arenavirus GPC domains, the generation of a consensus construct (C) or the sequential immunization with different Arenavirus GPCs (D). A vaccine strategically activating T cells might also be beneficial. A strategy for such a vaccine might be the generation of nanoparticles encompassing T cell activating peptides and presenting GPCs on their surface (E). Another approach would be to administer germline targeting immunogens that lead to the proliferation of naïve B cells that have specific B cell receptors after priming (F). Through so called shepherding immunizations and the final polishing immunization the desired B cells can be stimulated further.

The addition and removal of glycans, is a tool in rational vaccine design that enables the refocusing of immune responses and abrogates production of non-neutralizing or undesired antibody populations. This process is referred to as glycan masking and is based on the idea that non-relevant epitopes, are hidden by the addition of glycans, allowing the immune response to be refocused on vulnerable epitopes (Duan et al., 2018; Martina et al., 2023) (Fig. 6A). The introduction of these glycans requires an in depth knowledge of the structure of the antigen-antibody interactions, as otherwise protein miss-folding or unfavorable immune responses might arise. Additionally algorithms that predict glycosylation sites and sites that can easily mutate into glycosylation sites can aid in vaccine development (Gupta and Brunak, 2002; Chuang et al., 2012). The introduction of rationally designed glycans has also shown promising results in the avoidance of ADE for other viruses, like Dengue. It could be observed in animal models that the removal or masking of highly conserved Flavivirus regions in vaccines still led to a protective immune response but infection with other Flaviviruses like Zika was not exasperated in contrast to the control groups where significant ADE was observed (Martina et al., 2023; Lin et al., 2019; Ulrich et al., 2020). Computational modeling might be advantageous for the prediction of B cell epitopes that could potentially also be recognized poorly by antibodies originally directed against other Arenaviruses (Fischer et al., 2022). Glycan masking, however, limits the vaccine breadth by focusing the immune response on specific epitopes only. The breadth could potentially be enhanced by the inclusion of T cell epitopes that are presented on several Arenaviruses (Fig. 6E). This could be especially beneficial for LASV, as robust T cell responses seem to be indicative for survival during infection (Hallam et al., 2018b). Thus, it could be conceivable to use nanoparticles that encompass broadly reactive T cell epitope peptides and present GPCs on their surface. These epitopes could also be derived from other Arenavirus proteins such as Z that are oftentimes more conserved than the GPC (Capul, and de la Torre Juan Carlos, Buchmeier Michael J., 2011) (Fig. 6E). There epitopes could then be incorporated into vaccine candidates and tested experimentally.

### 7.3. Strategies for a pan-Arenavirus vaccine

Several approaches can be employed for the development of a pan-Arenavirus vaccine, such as the immunization with a chimeric or mosaic GPC, glycan masking of epitopes that are not conserved like the GP1 domain, immunization with an artificial consensus construct, immunization with a regimen comprised of different Arenavirus GPCs (Fig. 6) (Nachbagauer et al., 2021; Dirk et al., 2014; Xie et al., 2019; Wei et al., 2020b). A vaccine based on the latter approach, where non-human primates were immunized with a highly attenuated Arenavirus expressing the GPCs of either MACV, GTOV, SABV, JUNV or all NW Arenaviruses, administered as an equivalent mixture conferred sterile protection from NW Arenaviruses (Reynard et al., 2023). The protection from OW Arenavirus infection, however, was not shown and it is unlikely that cross neutralization to OW Arenaviruses takes place. It could be shown for both NW and OW Arenaviruses that the immunization of non-human primates with their respective nonpathogenic relatives, like Tacaribe-Virus or Mopeia virus, leads to cross-protecting immune responses (Ölschläger and Flatz, 2013; Kiley et al., 1979). Again, cross neutralization against viruses of the respective other group was not shown. Concluding the development of pan-Arenavirus vaccine poses a challenge, as Arenaviruses bind to a range receptors and their RBDs are positioned differently on the GPC. Another obstacle is the poorly characterized humoral immune response, which is necessary to determine targets for germline targeting or epitope-focusing. Nevertheless, promising results were obtained with structure-driven pan-Coronavirus vaccine design approaches (Williams et al., 2023; Lewitus et al., n.d.; Ong et al., 2020). These could potentially also support the development of a pan-Arenavirus vaccine. Still, it is likely that only the development of a pan-NW and a pan-OW vaccine is successful due to the profound differences between the viruses.

Another option in the development of a pan-Arenavirus vaccine could be the employment of germline targeting that has proven highly promising in the quest for an HIV vaccine (Steichen et al., 2016; Leggat et al., 2022). Germline targeting relies on the activation of naïve B cells that express certain germline B cell receptors that are progenitors of broadly neutralizing antibodies. These B cells will then be “shepherded” and “polished” by immunization with strategically designed immunogens ultimately leading to the generation of plasma cells that elicit these broadly neutralizing antibodies (Steichen et al., 2016; Leggat et al., 2022) (Fig. 6F). Most LASV neutralizing antibodies share the same germline heavy chain identity (VH1–2*02) and have a varied light chain identity just like broadly neutralizing HIV antibodies do (Li et al., n.d.; Robinson et al., 2016; Hastie et al., 2019). This could make them apt for being elicited by germline targeting vaccines. In contrast to HIV however, LASV antibodies are characterized by a low somatic hypermutation rate in comparison to the germline, with 10–15% whilst HIV broadly neutralizing antibodies are heavily mutated with rates up to 40% (supplement, Table 1). The comparatively low mutation rate of LASV antibodies argues, that they can arise relatively quickly and that B cells eliciting these antibodies do not require guiding or shepherding to produce a certain type of antibodies (Wu et al., 2010). Additionally, only few Arenavirus neutralizing antibodies have been identified thus far, mostly due to a lack of effort or availability of samples. It is therefore very likely, that antibodies with a different heavy and light chain usage might be found that neutralize more efficiently than the antibody set that is currently available.

The lack of antibodies also has implications for the analysis of the immunogen candidates since these oftentimes rely on a panel of antibodies, that so far has only been identified for LASV (Robinson et al., 2016). Thus using the LASV neutralizing and binding antibodies as scaffold for redesign to bind other OW and NW viruses could be a valid strategy to obtain more tools. Indeed, the redesign of antibodies has already been used successfully by Hastie et al., who redesigned the LASV antibody 18.5C to bind LCMV (Hastie et al., 2016). There are nowadays multiple computational options that allow for such approaches: RosettaAntibodyDesign (RAbD), Protein MPNN and protein language models (Dauparas et al., 2022; North et al., 2011; Adolf-Bryfogle et al., 2018; Singh, 2023; Schoeder et al., 2021). Another tool, that could be useful during immunogen design are mini binders, which have been identified by computational de novo design in the past for Influenza hemagglutinin and SARS-CoV-2 Spike. Strauch et al. for example, were able to design trimeric proteins that target the hemagglutinin receptor binding site and thereby neutralize Influenza (Strauch et al., 2017). To achieve this, a small protein, that binds to a single site on hemagglutinin, was designed with Rosetta. In a second design step it was modified to reassemble into a homo-oligomeric trimer that binds optimally to trimeric hemagglutinin. This approach could also be applied when designing small proteins for the neutralization of Arenaviruses. It could thus be possible to design a trimeric protein mimicking the binding of the LASV 8.9F antibody targeting three apical sites on the protein (Fig. 5A, B). It could also be feasible to design a protein mimicking the of LASV class iii or iv proteins that interact both with the GP1 and the GP2 domain and thereby neutralize (Fig. 5A, B). Mini binders could also be designed to interact with the SSP domain thereby inhibiting Arenavirus infectivity. Rosetta protocols were also successfully employed for the design of picomolar SARS-CoV-2 mini protein inhibitors that block the host-virus interaction (Cao et al., 2020). Novel methods, like RoseTTAFold diffusion (RFdiffusion) have also been used for the design of de novo binding proteins (Watson et al., 2023). Watson et al. used this artificial intelligence based method that is able to design novel unconditional and topology-constrained proteins for the development of mini binders that interact with hemagglutinin (Watson et al., 2022). Concluding, several methods are available and have successfully been employed in mini binder design of proteins. Since mini binders can be produced cost effectively, are highly stable and small they would be a great contribution to the Arenavirus diagnostics and therapeutics toolbox and should be investigated in depth.

## 8. Conclusion and outlook

Arenaviruses pose a significant threat to global health and many factors regarding their life cycle, mode of infection and structure remain elusive to this day. Hence, we propose, that many of the questions that arise during Arenavirus vaccine design can be answered by the employment of rational structure-based strategies. These could not only result in a potential Arenavirus vaccine but also in the development of a computational pipeline that can easily be transferred and applied to other viruses. A major challenge to these endeavors are the lack of known antibody interactions, especially for NW Arenaviruses, the broad and undefined receptor range and the lack of X-Ray or cryo-EM structures.

Furthermore, it will remain challenging to generate breadth over NW and OW Arenaviruses. Studies investigating concepts for vaccine design over both will provide evidence how a broad protection will be established. Whether this will use a molecular approach and redesign of a specific protein or multivalence in the vaccine will have to be determined.

Nevertheless, no point in time was more suitable to tackle this challenge as new methods especially coming from computational protein design are enabling a rational and rapid approach towards Arenavirus vaccine design.

## Supplementary Material

Supplement- Appendix A

## Figures and Tables

**Table 1:** Human pathogenic Arenaviruses.

*Old world (OW)*					
*Virus*	Region of origin	Reservoir	Primary host cell receptor	Secondary host cell receptor	References

*Lassa virus (LASV)*	West Africa	*Mastomys natalensis* and other mammals	α-Dystroglycan (α-DG)	lysosomal-associated membrane protein 1 (LAMP1)	(Brisse and Ly, 2019; Happi et al., 2022; Garry, 2023; Ly, 2017; Pennington and Lee, 2022; Katz et al., 2022)
*Lymphocytic choriomeningitis virus (LCMV)*	Worldwide	*Mus musculus*	α-DG	CD164	(Vilibic-Cavlek et al., 2021; Saito et al., 2023; Bakkers et al., 2022; Hastie et al., 2016)
*Lujo virus (LUJV)*	South Africa	unknown	neuropilin-2 (NRP2)	CD63	(Brisse and Ly, 2019; Simulundu et al., 2016; Cohen-Dvashi et al., 2018; Kunz and de la Torre, 2017)
*New World (NW)*					
*Junin virus (JUNV)*	South America	*Calomys musculinus, Calomys laucha*	Transferrin receptor-1 (TfR1)	unknown	(Linero et al., 2018; Grant et al., 2012; Radoshitzky et al., 2007; Clark et al., 2018; Sarute and Ross, 2017)
*Machupo virus (MACV)*	South America	*Calomys collosus*	TfR1	unknown	(Brisse and Ly, 2019; Sarute and Ross, 2017; Patterson et al., 2014; Radoshitzky et al., 2011; Ng et al., 2022)
*Guanarito virus (GOTV)*	South America	*Zygodontomys brevicauda*	TfR1	unknown	(Sarute and Ross, 2017; Marie-Laure et al., 2013; Silva-Ramos et al., 2022)
*Sabia virus (SABV)*	South America	unknown	TfR1	unknown	(Brisse and Ly, 2019; Sarute and Ross, 2017; Kerr et al., 2015)
*Chapare virus (CHAV)*	South America	*Oligoryzomys microtis*	TfR1	unknown	(Sarute and Ross, 2017; Loayza Mafayle et al., 2022; Gustavo et al., 2012)
*White water arroyo virus (WWAV)*	North America	*Neotoma albigula*	TfR1	unknown	(Sarute and Ross, 2017; Rhys et al., 2018; Min et al., 2014; Lele et al., 2003)

## Data Availability

No data was used for the research described in the article.
