# Supplementary material for: Strategies of rational and structure-driven vaccine design for Arenaviruses
Source: Infect Genet Evol. Author manuscript; Available in PMC 2025 Apr 21. (PMC12010953; doi:10.1016/j.meegid.2024.105626)
Supplement: Supplement- Appendix A [file NIHMS2069316-supplement-Supplement-_Appendix_A.docx]

**Supplement**

Table 1: neutralizing antibodies directed against Arenaviruses

The virus against which the mAbs were identified is highlighted in blue., n.d. not determined, all antibodies bind discontinuous, conformational epitopes

|  | **OW** | | | **NW** | |  |  |  |  |  | **glycosylation** | | |  | | |
| --- | --- | --- | --- | --- | --- | --- | --- | --- | --- | --- | --- | --- | --- | --- | --- | --- |
| **mAB** | **LASV** | **LCMV** | **LUJV** | **JUNV** | **MACV** | **LASV Strains** | **Origin** | **HC Mutation %** | **LC Mutation %** | **Putative epitope (amino acids)** | **Contact** | **Facilitating** | **Blocking** | **PDB** | **References** | |
| **10.4B** | + | + | - | n.d. | - | I, II, IV | human | 88.89 | 87.81 | 111-117 | n.d. | n.d. | n.d. | n.d. | (Robinson et al., 2016) |  |
| **18.5C-M30** | + | n.d. | n.d. | n.d. | n.d. | I, IV | engineered | n.d. | 92.55 | 66-72  364-371 | n.d. | n.d. | n.d. | 7UL7 | (Buck Tierra K. et al., 2022) |  |
| **12.1F** | + | + | - | n.d. | - | I, II, III, IV | human | 91.23 | 92.47 | 111-117 | LASV  N89 N109 | LASV  N89N109 | n.d. | 8EJH  7UOV | (Li et al., n.d.; Robinson et al., 2016) |  |
| **18.5C** | + | + | - | n.d. | - | II, III, IV | human | 88.89 | 92.55 | 66-72  364-371 | n.d. | n.d. | n.d. | 6P91 | (Buck Tierra K. et al., 2022; Hastie et al., 2019; Robinson et al., 2016) |  |
| **19.7E** | + | - | - | n.d. | - | I, II, IV | human | 92.36 | 94.62 | 111-117 | n.d. | LASV  N109N167 | n.d. | 8EJI | (Robinson et al., 2016) |  |
| **2.9D** | + | + | - | n.d. | - | I, II, III, IV | human | 93.06 | 98.99 | 66-72  364-371 | n.d. | n.d. | n.d. | n.d. | (Robinson et al., 2016) |  |
| **25.10C** | + | - | - | n.d. | - | pan-LASV | human | 91.32 | 93.19 | 62-68  270-278 | LASV  N79 N99 N224 | n.d. | n.d. | 7UDS  7TYV | (Buck Tierra K. et al., 2022; Robinson et al., 2016) |  |
| **25.6A** | + | + | - | n.d. | - | I, II, III, IV | human | 65.26 | 93.75 | 66-72  364-371 | n.d. | n.d. | n.d. | 6P95 | (Buck Tierra K. et al., 2022; Hastie et al., 2019; Robinson et al., 2016) |  |
| **36.1F** | + | - | - | n.d. | - | (II), IV | human | 87.02 | 93.62 | 62-68  270-278 | n.d. | n.d. | n.d. | n.d. | (Buck Tierra K. et al., 2022; Robinson et al., 2016) |  |
| **36.9F** | + | + | - | n.d. | - | I, II, III, IV | human | 91.32 | 63.64 | 66-72  364-371 | n.d. | n.d. | n.d. | n.d. | (Robinson et al., 2016) |  |
| **37.2D** | + | + | - | n.d. | - | I, II, III, IV | human | 89.24 | 94.27 | 66-72  364-371 | LASV  N390N395 | n.d. | LASV  N390  N395 | 7UOT  7UOV | (Li et al., n.d.; Robinson et al., 2016) |  |
| **37.2G** | + | + | - | n.d. | - | II, III, IV | human | 93.75 | 96.45 | 66-72  364-371 | n.d. | n.d. | n.d. | n.d. | (Robinson et al., 2016) |  |
| **37.7H** | + | + | - | n.d. | - | I, II, III, IV | human | 66.32 | 94.1 | 66-72  364-371 | LASV  N390N395 | n.d. | LASV  N390  N395 | 5VK2 | (Buck Tierra K. et al., 2022; Hastie et al., 2019; Robinson et al., 2016) |  |
| **8.11G** | + | - | - | n.d. | - | I, II, III, IV | human | 90.53 | 88.53 | 62-68  270-278 | n.d. | n.d. | n.d. | n.d. | (Gorman et al., 2024; Robinson et al., 2016) |  |
| **8.9F** | (+) | - | - | n.d. | - | I, II, III, IV | human | 92.01 | 94.1 | 125-150  256-259 | LASV  N119 | LASV  N119 | n.d. | 7UOT | [(Li et al., n.d.; Robinson et al., 2016)](https://doi.org/10.1126/scitranslmed.abq0991) |  |
| **9.8A** | + | - | - | n.d. | - | I, II, III, IV | human | 95.83 | 96.42 | 66-72  364-371 | n.d. | n.d. | n.d. | n.d. | (Robinson et al., 2016) |  |
| **NE13** | + | - | - | n.d. | - | II, III, IV | human | 96.53 | 94.68 | 66-72  364-371 | n.d. | n.d. | n.d. | n.d. | (Robinson et al., 2016) |  |
| **LAVA01** | + | n.d. | n.d. | n.d. | n.d. | II, III, IV | rabbit | n.d. | n.d. | 152-156  163-167  297-358 | LASV  N109 N390 | LASV  N395 | LASV  N390 | 7SGF | (Brouwer et al., 2022) |  |
| **18.5C-M28** | + | + | n.d. | n.d. | n.d. | n.d. | engineered | n.d. | n.d. | 411,414,281,301 | n.d. | n.d. | n.d. | 8DMH | (Moon-Walker et al., 2023) |  |
| **KL25** | n.d. | + | n.d. | n.d. | n.d. | n.d. | mouse | n.d. | n.d. | 119 | n.d. | n.d. | n.d. | n.d. | (Bruns et al., 1983; Hangartner et al., 2006) |  |
| **GD01** | - | - | - | + | - | n.d. | mouse | n.d. | n.d. | 122,123 | n.d. | n.d. | n.d. | 5EN2 | (Sanchez et al., 1989) |  |
| **QC03** | - | - | - | + | - | n.d. | mouse | n.d. | n.d. | 122,123 | n.d. | n.d. | n.d. | n.d. | (Sanchez et al., 1989) |  |
| **OD01** | - | - | - | + | - | n.d. | mouse | n.d. | n.d. | n.d. | n.d. | n.d. | n.d. | 5NUZ | (Sanchez et al., 1989) |  |
| **GB03** | - | - | - | + | - | n.d. | mouse | n.d. | n.d. | n.d. | n.d. | n.d. | n.d. | n.d. | (Sanchez et al., 1989) |  |
| **J86** | n.d. | n.d. | n.d. | + | n.d. | n.d. | mouse | n.d. | n.d. | 122,123 | n.d. | n.d. | n.d. | n.d. | (Pan et al., 2018) |  |
| **J88** | n.d. | n.d. | n.d. | + | n.d. | n.d. | mouse | n.d. | n.d. | 122,123 | n.d. | n.d. | n.d. | n.d. | (Pan et al., 2018) |  |
| **J99** | n.d. | n.d. | n.d. | + | n.d. | n.d. | mouse | n.d. | n.d. | 122,123 | n.d. | n.d. | n.d. | n.d. | (Pan et al., 2018) |  |
| **J100** | n.d. | n.d. | n.d. | + | n.d. | n.d. | mouse | n.d. | n.d. | 122,123 | n.d. | n.d. | n.d. | n.d. | (Pan et al., 2018) |  |
| **J108** | n.d. | n.d. | n.d. | + | n.d. | n.d. | mouse | n.d. | n.d. | 122,123 | n.d. | n.d. | n.d. | n.d. | (Pan et al., 2018) |  |
| **CR1-07** | - | n.d. | n.d. | + | + | n.d. | human | 86.8 | 93.5 | 115-127, 165-174 | n.d. | n.d. | n.d. | 5W1G, 5W1M | (Clark et al., 2018) |  |
| **CR1-28** | - | n.d. | n.d. | + | + | n.d. | human | 96.3 | 98.5 | 111-117,216 | n.d. | n.d. | n.d. | 5W1K | (Clark et al., 2018) |  |
| **JUN1** | n.d | n.d | n.d | + | - | n.d | mouse | 97.3 | 92.5 | 111,113,170,171 | n.d. | n.d. | n.d. | 7QU2 | (Ng Weng M. et al., 2022) |  |
| **KL-MM-1B6** | - | n.d. | n.d. | n.d. | + | n.d. | mouse | n.d. | n.d. | 226 | n.d. | n.d. | n.d. | n.d. | (Amanat Fatima et al., 2020) |  |
| **KL-MM-1F9** | + | n.d. | n.d. | n.d. | + | n.d. | mouse | n.d. | n.d. | 226, 169, 170 | n.d. | n.d. | n.d. | n.d. | (Amanat Fatima et al., 2020) |  |
| **KL-MM-2C8** | - | n.d. | n.d. | n.d. | + | n.d. | mouse | n.d. | n.d. | 226 | n.d. | n.d. | n.d. | n.d. | (Amanat Fatima et al., 2020) |  |
| **KL-MM-4C12** | + | n.d. | n.d. | n.d. | + | n.d. | mouse | n.d. | n.d. | 170, 174, 226 | n.d. | n.d. | n.d. | n.d. | (Amanat Fatima et al., 2020) |  |
| **KL-MM-4G7** | + | n.d. | n.d. | n.d. | + | n.d. | mouse | n.d. | n.d. | 226 | n.d. | n.d. | n.d. | n.d. | (Amanat Fatima et al., 2020) |  |
| **MAC1** | n.d | n.d | n.d | - | + | n.d | mouse | 97.9 | 95.0 | 117-122, 165-171, 226 | n.d. | n.d. | n.d. | 7QU1 | (Ng Weng M. et al., 2022) |  |

**References**

Amanat Fatima, Duehr James, Huang Cheng, Paessler Slobodan, Tan Gene S., Krammer Florian, 2020. Monoclonal Antibodies with Neutralizing Activity and Fc-Effector Functions against the Machupo Virus Glycoprotein. J. Virol. 94, 10.1128/jvi.01741-19. https://doi.org/10.1128/jvi.01741-19

Brouwer, P.J.M., Antanasijevic, A., Ronk, A.J., Müller-Kräuter, H., Watanabe, Y., Claireaux, M., Perrett, H.R., Bijl, T.P.L., Grobben, M., Umotoy, J.C., Schriek, A.I., Burger, J.A., Tejjani, K., Lloyd, N.M., Steijaert, T.H., van Haaren, M.M., Sliepen, K., de Taeye, S.W., van Gils, M.J., Crispin, M., Strecker, T., Bukreyev, A., Ward, A.B., Sanders, R.W., 2022. Lassa virus glycoprotein nanoparticles elicit neutralizing antibody responses and protection. Cell Host Microbe 30, 1759-1772.e12. https://doi.org/10.1016/j.chom.2022.10.018

Bruns, M., Cihak, J., Müller, G., Lehmann-Grube, F., 1983. Lymphocytic choriomeningitis virus. VI. Isolation of a glycoprotein mediating neutralization. Virology 130, 247–251. https://doi.org/10.1016/0042-6822(83)90135-6

Buck Tierra K., Enriquez Adrian S., Schendel Sharon L., Zandonatti Michelle A., Harkins Stephanie S., Li Haoyang, Moon-Walker Alex, Robinson James E., Branco Luis M., Garry Robert F., Saphire Erica Ollmann, Hastie Kathryn M., 2022. Neutralizing Antibodies against Lassa Virus Lineage I. mBio 13, e01278-22. https://doi.org/10.1128/mbio.01278-22

Clark, L.E., Mahmutovic, S., Raymond, D.D., Dilanyan, T., Koma, T., Manning, J.T., Shankar, S., Levis, S.C., Briggiler, A.M., Enria, D.A., Wucherpfennig, K.W., Paessler, S., Abraham, J., 2018. Vaccine-elicited receptor-binding site antibodies neutralize two New World hemorrhagic fever arenaviruses. Nat. Commun. 9, 1884. https://doi.org/10.1038/s41467-018-04271-z

Gorman, J., Cheung, C.S.-F., Duan, Z., Ou, L., Wang, M., Chen, X., Cheng, C., Biju, A., Sun, Y., Wang, P., Yang, Y., Zhang, B., Boyington, J.C., Bylund, T., Charaf, S., Chen, S.J., Du, H., Henry, A.R., Liu, T., Sarfo, E.K., Schramm, C.A., Shen, C.-H., Stephens, T., Teng, I.-T., Todd, J.-P., Tsybovsky, Y., Verardi, R., Wang, D., Wang, S., Wang, Z., Zheng, C.-Y., Zhou, T., Douek, D.C., Mascola, J.R., Ho, D.D., Ho, M., Kwong, P.D., 2024. Cleavage-intermediate Lassa virus trimer elicits neutralizing responses, identifies neutralizing nanobodies, and reveals an apex-situated site-of-vulnerability. Nat. Commun. 15, 285. https://doi.org/10.1038/s41467-023-44534-y

Hangartner, L., Zellweger, R.M., Giobbi, M., Weber, J., Eschli, B., McCoy, K.D., Harris, N., Recher, M., Zinkernagel, R.M., Hengartner, H., 2006. Nonneutralizing antibodies binding to the surface glycoprotein of lymphocytic choriomeningitis virus reduce early virus spread. J. Exp. Med. 203, 2033–2042. https://doi.org/10.1084/jem.20051557

Hastie, K.M., Cross, R.W., Harkins, S.S., Zandonatti, M.A., Koval, A.P., Heinrich, M.L., Rowland, M.M., Robinson, J.E., Geisbert, T.W., Garry, R.F., Branco, L.M., Saphire, E.O., 2019. Convergent Structures Illuminate Features for Germline Antibody Binding and Pan-Lassa Virus Neutralization. Cell 178, 1004-1015.e14. https://doi.org/10.1016/j.cell.2019.07.020

Li, H., Buck, T., Zandonatti, M., Yin, J., Moon-Walker, A., Fang, J., Koval, A., Heinrich, M.L., Rowland, M.M., Diaz Avalos, R., Schendel, S.L., Parekh, D., Zyla, D., Enriquez, A., Harkins, S., Sullivan, B., Smith, V., Chukwudozie, O., Watanabe, R., Robinson, J.E., Garry, R.F., Branco, L.M., Hastie, K.M., Saphire, E.O., n.d. A cocktail of protective antibodies subverts the dense glycan shield of Lassa virus. Sci. Transl. Med. 14, eabq0991. https://doi.org/10.1126/scitranslmed.abq0991

Moon-Walker, A., Zhang, Z., Zyla, D.S., Buck, T.K., Li, H., Diaz Avalos, R., Schendel, S.L., Hastie, K.M., Crotty, S., Saphire, E.O., 2023. Structural basis for antibody-mediated neutralization of lymphocytic choriomeningitis virus. Cell Chem. Biol. 30, 403-411.e4. https://doi.org/10.1016/j.chembiol.2023.03.005

Ng Weng M., Sahin Mehmet, Krumm Stefanie A., Seow Jeffrey, Zeltina Antra, Harlos Karl, Paesen Guido C., Pinschewer Daniel D., Doores Katie J., Bowden Thomas A., 2022. Contrasting Modes of New World Arenavirus Neutralization by Immunization-Elicited Monoclonal Antibodies. mBio 13, e02650-21. https://doi.org/10.1128/mbio.02650-21

Pan, X., Wu, Y., Wang, W., Zhang, L., Xiao, G., 2018. Novel neutralizing monoclonal antibodies against Junin virus. Antiviral Res. 156, 21–28. https://doi.org/10.1016/j.antiviral.2018.06.002

Robinson, J.E., Hastie, K.M., Cross, R.W., Yenni, R.E., Elliott, D.H., Rouelle, J.A., Kannadka, C.B., Smira, A.A., Garry, C.E., Bradley, B.T., Yu, H., Shaffer, J.G., Boisen, M.L., Hartnett, J.N., Zandonatti, M.A., Rowland, M.M., Heinrich, M.L., Martínez-Sobrido, L., Cheng, B., de la Torre, J.C., Andersen, K.G., Goba, A., Momoh, M., Fullah, M., Gbakie, M., Kanneh, L., Koroma, V.J., Fonnie, R., Jalloh, S.C., Kargbo, B., Vandi, M.A., Gbetuwa, M., Ikponmwosa, O., Asogun, D.A., Okokhere, P.O., Follarin, O.A., Schieffelin, J.S., Pitts, K.R., Geisbert, J.B., Kulakoski, P.C., Wilson, R.B., Happi, C.T., Sabeti, P.C., Gevao, S.M., Khan, S.H., Grant, D.S., Geisbert, T.W., Saphire, E.O., Branco, L.M., Garry, R.F., 2016. Most neutralizing human monoclonal antibodies target novel epitopes requiring both Lassa virus glycoprotein subunits. Nat. Commun. 7, 11544. https://doi.org/10.1038/ncomms11544

Sanchez, A., Pifat, D.Y., Kenyon, R.H., Peters, C.J., McCormick, J.B., Kiley, M.P., 1989. Junin Virus Monoclonal Antibodies: Characterization and Cross-reactivity with Other Arenaviruses. J. Gen. Virol. https://doi.org/10.1099/0022-1317-70-5-1125
